# Catarrh

**Published:** 1890-11

**Authors:** 


### CATARRH.

There are several distinct forms of catarrh, which may be classed under two general heads, the dry or non-discharging, and the mucous or flowing catarrh, of which the latter is by far the more prevalent.

A cold in the head, strictly speaking, is a catarrh, and if suffered to run its course without interruption, may assume all the worst features of this troublesome disease, and finally become so thoroughly seated as to occasion no little difficulty in its dislodgment. It first attacks the mucous membrane of the nasal apertures, inflaming it and causing a continuous watery flow, thence extending downward to the air passages leading to the chest, causing constant irritation, and finally settles upon the lungs with a deadly grip. Its worst symptoms are attended with headache, impaired hearing, sight and memory, loss of appetite and general debility. We have known cases where the loss of smell as well as taste were attributable to this cause.

Ozœna was the Greek name given to this disease, which is a torpid ulcer in the deeper portions of the nasal passages, with more or less offensive discharges. Its progress should be vigorously resisted with curative remedies before it has time to fasten upon the lower and more sensitive air passages leading to the chest.

There are a great many specifics which are claimed to be efficacious in its treatment, but a capable physician should by all means be consulted before a regular course of treatment is entered upon.

